# A case of bilateral thalamic infarct complicating tuberculous meningoencephalitis

**DOI:** 10.11604/pamj.2019.33.2.13327

**Published:** 2019-05-06

**Authors:** Jehanne Aasfara, Wafa Regragui, Loubna El Ouardi, El Hachmia Ait Ben Haddou, Ali Benomar, Mohammed Yahyaoui

**Affiliations:** 1 Department of Neurology and Neurogenetics, Hôpital des Spécialités ONO, CHU Rabat-Salé, Morocco; 2 Faculty of Medicine and Pharmacy, University Mohamed V Souissi, Rabat, Morocco

**Keywords:** Tuberculosis, meningoencephalitis, stroke, bithalamic, movement disorders

## Abstract

Ischemic stroke can result from multiple etiologies. It can also be a complication of tuberculous meningoencephalitis and determine its outcome. stroke secondary to tuberculous meningoencephalitis, occurs in 30% cases in the basal ganglia region, unusually in the thalamus. The mechanism of stroke in this condition is vasculitis. We report an unusual case of bilateral thalamic infarcts complicating tuberculous meningoencephalitis. Ischemic stroke in tuberculous meningoencephalitis is unpredictable with poor prognosis despite antituberculous drug treatment, emphasising the importance of primary prevention, particularly in tuberculosis endemic areas.

## Introduction

Ischemic stroke in tuberculous meningoencephalitis (TME), occurs in the basal ganglia region, internal capsule and rarely in the thalamus [1, 2]. Bilateral infarct is exceptional [3]. We report a rare case of bilateral thalamic infarcts complicating tuberculous meningoencephalitis.

## Patient and observation

**Figure 1:**
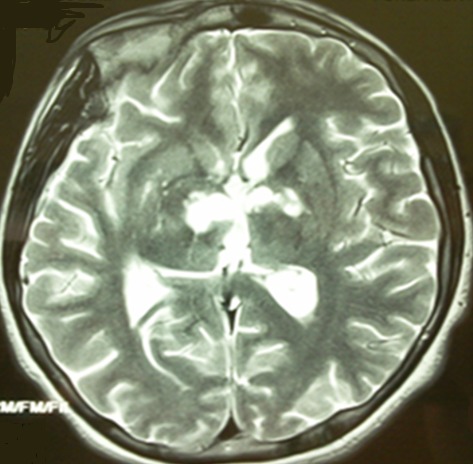
Cerebral MRI axial T2 weighted image: abnormal signal in bilateral anterior thalami and right capsular genu

**Figure 2:**
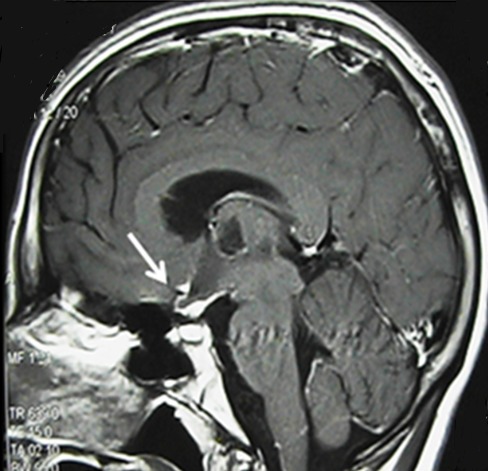
Cerebral MRI sagittal T1 weighted image: hypothalamic enhancement after contrast administration

A 15 year-old male, with no medical history, presented with febrile coma. Cerebral CT scan was normal and cerebrospinal fluid (CSF) analysis showed lymphocytic pleocytosis (160cells/mm^3^), protein concentration up to 0.97g/l, glucose at 1.6mmol/l (CSF/blood glucose < 0.5) and hyponatremia in serum analysis. The patient was treated by intravenous steroids for three days and antituberculous drugs. Fifteen days later; the patient developed agitated confusion with left hemiparesis and was referred to our department for further investigation and care. Neurological examination revealed fluctuant altered consciousness, left hemiparesis, axial twisting movements on the left upper limb with severe dystonia. Brain MRI found bilateral anterior and medial thalami ischemic lesions extended to internal globus pallidus and subthalamic nucleus with hypothalamic enhancement (Figure 1 and Figure 2). CSF analysis showed 36 cells/mm^3^ (92% lymphocytes), protein at 1.19g/l, glucose 2mmol/l. Mycobacterium tuberculosis Culture in CSF and 3 sputum examinations were negative. Plasma Angiotensin I-converting enzyme was normal. Thoracic CT scan showed bilateral asymmetric mediastinal lymphadenopathy with hypodense centers evoking caseous necrosis. Bronchoscopy with bronchial biopsies revealed no specific bronchial mucosal inflammation. Bronchoalveolar lavage showed polynucleosis. The diagnosis of bilatreal thalamic infarct complicating TME was made. Antituberculous drugs were maintained and oral corticosteroids administered with symptomatic treatment based on trihexyphenidyle, diazepam, botulinum toxine and fluoxetine. The outcome was favorable concerning hemiparesis, mutism and movement disorders but the patient was disabled by left segmental dystonia and showed some behavioral disorders that resolved with haloperidol. Three months later, cerebral MRI showed nearly complete resolution of signal abnormalities but neuropsychological assessment revealed thalamic dementia.

## Discussion

Our patient illustrates vascular complication of tuberculous meningoencephalitis. Cerebral vasculitis is a rare cause of blood vessel walls inflammation. Stroke occurs in 15-57% of tuberculous meningitis cases [4]. The primary mechanism of stroke in tuberculous meningoencephalitis is inflammation of both large and small vessels walls [5]. “tubercular zone” supplied by medial striate and thalamo- perforating arteries, is the most frequently involved area. It includes the caudate, anterior thalamus, anterior limb and genu of the internal capsule [4-6]. Bilateral thalamic infarcts represented approximately 0.6% of all cerebral infarctions and result of Percheron artery occluision [7]. In our knowledge, bithalamic infarct has been reported in only two cases of tuberculous meningoencephalitis [8, 9]. It has been previously demonstrated that early strokes in TME are mediated by vasospasm and later strokes by proliferative intimal disease. stroke occur in our case, fifteen days after initiation of antituberculous drugs. It is likely immune-mediated reaction resulting of host-organism interaction. Indeed, vascular complications are more commonly seen in chronic meningoencephalitis and continue to develop during the first weeks of treatment (9%) [10]. In some cases, stroke can be concomitant to corticosteroid decrease which was the case of our patient. Neverthless, the preventive role of corticosteroids remains a subject of controversy [7]. Movement disorders in TME have been reported in up to 16.6% cases. Basal ganglia infarcts are presumably one of their mechanisms. There is poor correlation between neurological findings and focal lesion on imaging studies [11]. Dystonia is commonly due to frontal or parietal lesions and rarely to basal ganglia lesions [12]. In our patient, we can qualify axial twisting mouvements of hand as “thalamic hand” and dystonia of proximal left upper limb as “thalamic contracture.” Thalamic contracture is induced by posterior thalamic lesions whereas they were on anterior nuclei in our patient. We can explain these mouvement disorders by extensive bilateral lesions wich disrupted basal ganglia circuit. The poor outcome of our patient is likely due to cognitive impairment and focal dystonia secondary to multiple basal ganglia infarcts wich reported to be associated with poor prognosis than single infarcts (46.7% versus 32%) [13].

## Conclusion

Ischemic stroke in tuberculous meningoencephalitis is unpredictable with poor prognosis despite early treatment, especially in multiple infarcts, suggesting the importance of primary prevention, particularly in tuberculosis endemic areas.

## Competing interests

The authors declare no competing interests.
